# Giant coral reef fishes display markedly different susceptibility to night spearfishing

**DOI:** 10.1002/ece3.4501

**Published:** 2018-09-12

**Authors:** Alan R. Pearse, Richard J. Hamilton, John Howard Choat, John Pita, Glenn Almany, Nate Peterson, Grant S. Hamilton, Erin E. Peterson

**Affiliations:** ^1^ ARC Centre of Excellence for Mathematical and Statistical Frontiers (ACEMS) Queensland University of Technology Brisbane QLD Australia; ^2^ The Nature Conservancy Asia Pacific Resource Centre South Brisbane QLD Australia; ^3^ ARC Centre of Excellence for Coral Reef Studies James Cook University Townsville QLD Australia; ^4^ College of Science and Engineering James Cook University Townsville QLD Australia; ^5^ The Nature Conservancy Isabel Environmental Office Buala Isabel Province Solomon Islands; ^6^ CRIOBE – USR 3278 CNRS–EPHE–UPVD Laboratoire d'Excellence “CORAIL” Perpignan Cedex France; ^7^ School of Earth, Environmental and Biological Sciences Queensland University of Technology Brisbane QLD Australia; ^8^ The Institute for Future Environments Queensland University of Technology Brisbane QLD Australia

**Keywords:** bumphead parrotfish, humphead wrasse, night spearfishing, susceptibility

## Abstract

The humphead wrasse (*Cheilinus undulatus*) and bumphead parrotfish (*Bolbometopon muricatum*) are two of the largest, most iconic fishes of Indo‐Pacific coral reefs. Both species form prized components of subsistence and commercial fisheries and are vulnerable to overfishing. *C. undulatus* is listed as Endangered and *B. muricatum* as Vulnerable on the IUCN Red List of Threatened Species. We investigated how night spearfishing pressure and habitat associations affected both species in a relatively lightly exploited setting; the Kia fishing grounds, Isabel Province, Solomon Islands. We used fisheries‐independent data from underwater visual census surveys and negative binomial models to estimate abundances of adult *C. undulatus* and *B. muricatum* as a function of spearfishing pressure and reef strata. Our results showed that, in Kia, night spearfishing pressure from free divers had no measurable effect on *C. undulatus* abundances, but abundances of *B. muricatum* were 3.6 times lower in areas of high spearfishing pressure, after accounting for natural variations due to habitat preferences. It is likely the species’ different nocturnal aggregation behaviors, combined with the fishers’ use of night spearfishing by spot‐checking underpin these species’ varying susceptibility. Our study highlights that *B. muricatum* is extremely susceptible to night spearfishing; however, we do not intend to draw conservation attention away from *C. undulatus*. Our data relate only to the Kia fishing grounds, where human population density is low, the spot‐checking strategy is effective for reliably spearing large numbers of fish, particularly *B. muricatum*, and fisheries have only recently begun to be commercialized; such conditions are increasingly rare. Instead, we recommend that regional managers assess the state of their fisheries and the dynamics affecting the vulnerability of the fishes to fishing pressure based on local‐scale, fisheries‐independent data, where resources permit.

## 1 INTRODUCTION

The humphead wrasse (*Cheilinus undulatus*) and the bumphead parrotfish (*Bolbometopon muricatum*) are two of the largest coral‐reef fish in the Indo‐Pacific (Donaldson & Dulvy, 2004; Hamilton & Choat, 2012; Sadovy et al., 2003). Both species are labrids that grow to over 1 m in length and live in excess of 30 years (Andrews, Choat, Hamilton, & DeMartini, 2015; Choat, Davies, Ackerman, & Mapstone, 2006; Westneat & Alfaro, 2005). *C. undulatus* feeds on fish, molluscs, and echinoderms and has powerful pharyngeal dentition for crushing its prey (Colin & Sadovy de Mitcheson, 2012). Conversely, *B. muricatum* is a major bioeroder on coral reefs (Bellwood, Hoey, & Choat, 2003). It scavenges protein by consuming sessile animals (including coral), detritus, and endolithic autotrophs from shallow reef surfaces exposed to wave action, which is processed in the pharyngeal mill before digestion (Hamilton & Choat, 2012). Both species are inactive at night; a characteristic of labrids.

Significant declines in population density have been observed in both species over the last 30 years, which have been attributed to high levels of fishing to supply local and international markets (Fenner, 2014; Kindsvater, Reynolds, Savody de Mitcheson, & Mangel, 2017; Lavides et al., 2016). For example, Lavides et al. (2016) reported that, due to excess fishing pressure, the mean perceived biomass of *B. muricatum* and *C. undulatus* declined by 82% and 88%, respectively between the 1950s and 2014 in five regions of the Philippines. *B. muricatum* is sold as dead fish and is either speared at night or captured in nets during the day (Dulvy & Polunin, 2004; Hamilton & Choat, 2012), while *C. undulatus* is typically caught on handlines during the day or via diurnal or nocturnal spearfishing (Colin & Sadovy de Mitcheson, 2012; Hamilton, Giningele, Aswani, & Ecochard, 2012; Lindfield, McIlwain, & Harvey, 2014). The high value of *C. undulatus* and its ability to be captured in hook‐and‐line fisheries makes it a prime target of the live‐reef food‐fish trade (Sadovy et al., 2003; Zgliczynski et al., 2013). As a consequence of declining global populations, *C. undulatus* was listed as Endangered on the International Union for Conservation of Nature (IUCN) Red List of Threatened Species and added to Appendix II of the Convention on International Trade in Endangered Species of Wild Fauna and Flora in 2003 (CITES; Vincent, Sadovy de Mitcheson, Fowler, & Lieberman, 2014). *B. muricatum* was listed as Vulnerable on the IUCN Red List of Threatened Species in 2007 (Zgliczynski et al., 2013). However, the status of both species in the IUCN Red List of Threatened Species is presently under review (J.H. Choat, personal communication).

Night spearfishing is common throughout the Pacific (Gillett & Moy, 2006; Lindfield et al., 2014). Night spearfishers typically free‐dive with the aid of fins, mask, snorkel, a rubber powered spear, and an underwater flashlight. Small‐scale commercial fishers prefer night over daytime spearfishing because resting fish are easier to approach and spear, resulting in larger catches (Hamilton et al., 2012). In recent decades, considerable concern has been raised over the ease with which large iconic species such as *C. undulatus*,* B. muricatum,* and groupers can be overfished with night spearfishing (Gillett & Moy, 2006; Hamilton et al., 2012; Lindfield et al., 2014).

For *B. muricatum*, night spearfishing has been shown to rapidly deplete local populations once markets for this species develop (Dulvy & Polunin, 2004; Hamilton & Choat, 2012; Hamilton et al., 2016; Kobayashi et al. 2011). The vulnerability of *B. muricatum* relates to its nocturnal behavior. This species sleeps in schools in shallow water at highly predicable locations. Although *B. muricatum* sometimes sleep in cave systems in passage environments, they are typically found resting on the sand adjacent to corals, which makes them easy to locate (Hamilton, 2005). It has been suggested this nocturnal aggregating behavior coupled with their predictable resting locations causes hyperstability in *B. muricatum* fisheries, and may explain the dramatic collapse of *B. muricatum* fisheries across the Pacific (Hamilton et al., 2016). This vulnerability has also been observed in other aggregation fisheries (Sadovy & Domeier, 2005; Sadovy de Mitcheson & Erisman, 2012), such as the Atlantic cod fishery, where shoaling behavior led to increased catch rates even as population numbers dramatically declined (Hutchings, 1996).

Night spearfishing has also been implicated in the demise of *C. undulatus* populations (Colin & Sadovy de Mitcheson, 2012), particularly when night spearfishing is conducted on SCUBA (Lindfield et al., 2014). However, several aspects of *C. undulatus* nocturnal behavior suggest it may be less vulnerable to night spearfishing than *B. muricatum*. For example, it does not form nocturnal aggregations, and unlike *B. muricatum,* it frequently sleeps in caves and crevices within the reef matrix, making it harder to detect (Sadovy et al., 2003).

In this study, we had the unique opportunity to undertake an intensive, systematic evaluation of the effects of aggregation behavior on the susceptibility to fishing pressure for two closely related species, within one local fishery. We used underwater visual census (UVC) data and information on historical spearfishing pressure from free‐divers to explore the vulnerability of *C. undulatus* and *B. muricatum* to night spearfishing in the Kia fishing grounds in Isabel Province, Solomon Islands. At the time of this study, the extensive lagoon and outer reef systems of the Kia region supported abundant populations of *C. undulatus* and *B. muricatum*, even though free‐diving spearfishermen had operated a small‐scale, commercial night‐time spearfishery there since 2001 (Hamilton et al., 2016). The primary reasons for selecting this study site were fourfold: (a) the Kia region supports extensive reefs, low human population and limited market outlets, thus representing a lightly exploited region relative to many other areas of the Coral triangle; (b) fishery dependent and fishery independent biological data were available for both species; (c) the local fishery center provided data on catch rates and methods of fishing over significant time periods; and (d) the Kia House of Chiefs retain traditional ownership of the Kia fishing grounds and have the ability to implement management measures for both species within their customary fishing grounds. Thus, our aim was to use fisheries‐independent data to assess whether the abundances of adult *C. undulatus* and *B. muricatum* were related to fishing pressure, after accounting for the effects of reef strata (i.e., habitat) preferences.

## 2 MATERIALS AND METHODS

### 2.1 Study area, experimental design and data collection

**Figure 1:**
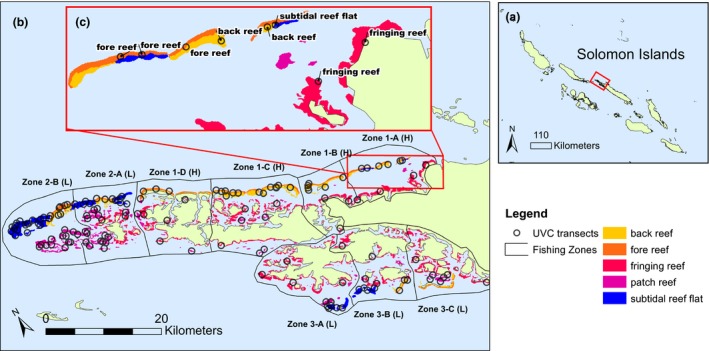
Map of the study area in the (a) Solomon Islands. The underwater visual census (UVC) surveys took place around (b) Kia, Santa Isabel, Solomon Islands in zones of high fishing pressure (H) and low fishing pressure (L). (c) Zone 1‐A, which includes the single subtidal reef transect in the zones of high fishing pressure

The study area is located in Kia District, Santa Isabel, Solomon Islands (Figure 1). It is approximately 1,250 km^2^ in size, with Kia being the largest community in the region. The majority of Kia district residents live a subsistence‐based lifestyle and retain customary ownership of their land and shallow seas. Kia inhabitants generate income through a combination of local‐scale agriculture and fishing, as well as royalties from commercial logging (Hamilton et al., 2017; Peterson, Hamilton, Pita, Atu, & James, 2012). In addition, two fisheries centers were operating in Kia District at the time this study was conducted. The Bahana Provincial Fisheries Centre (BPFC) had been operating in Kia since 2000, buying locally sourced fish for domestic export to the capital, Honiara. A private fishery also began operating in the nearby community of Babahairo in 2012 (Hamilton et al., 2016).

The environment is characterized by complex reefs with mangrove and coastal forests. Five major reef types are represented in the study area: back, fore, fringing, and patch reefs, as well as subtidal reef flats. Reefs were demarcated using maps from the Millenium Coral Reef Mapping Project (Andrefouet et al., 2006; see Supporting Information Appendix S1 for definitions). We also divided the study area into nine fishing zones based on interviews with 22 expert spearfishermen from the Kia district (Figure 1). The spearfishermen unanimously agreed Zones 1A‐D had historically experienced the greatest spearfishing pressure because the outer reefs are in close proximity to the BPFC. Zones 2A‐B and 3A‐C were further afield so there was less fishing activity due to fuel costs. In addition, few Kia fishermen had customary rights to Zones 3A‐C, which further reduced fishing pressure. Each zone has a length of at least 10 km, which encompasses the reported home ranges of *C. undulatus* and *B. muricatum* (Green et al., 2015).

In 2012, we performed 146 UVC surveys on the reef surrounding Kia (Fig. 1). UVC sites were selected prior to the fieldwork using a Generalized Random Tessellation Survey (GRTS; Stevens & Olsen, 2004), which accounted for differences in the total area of each reef type. The UVC transects consisted of 20‐min timed swims (Choat & Pears, 2003) and were conducted on SCUBA at depths between 2 and 12 m. Transects ranged between 174 and 848 m in length due to varying current speeds and were usually 20 m wide (126 of 146 transects). There were 20 transects where visibility fell below 10 m and these ranged between 8 and 16 m in width. Consequently, the total transect areas varied between 2,400 and 16,960 m^2^. SCUBA divers worked in pairs, with one diver swimming for 20 min with the prevailing current recording the size of all adult *C. undulatus* (≥35 cm total length) and *B. muricatum* (≥65 cm total length) sighted within the transect boundaries, while the other diver followed and towed a geographic positioning system (GPS) device along the surface. The divers conducting the UVC surveys were trained in fish identification and estimated the total length of each fish by eye. Note that we did not observe differences in the species’ responses to divers, or behavioral differences in high and low fishing‐pressure zones within species. Please see Hamilton et al. (2016) for additional information about the field surveys.

### 2.2 Statistical analyses

We started with an exploratory analysis of the data to (a) identify potential relationships between *B. muricatum* and *C. undulatus* abundance and the predictors (i.e., reef strata, fishing pressure, latitude, longitude) and (b) assess collinearity in the predictors. The exploratory analysis included numerical summaries of fish abundance, fish abundance within reef strata, and fish abundance by fishing pressure, as well as plots showing the relationship between them. We also used the generalized variance inflation factor (GVIF; Fox & Monette, 1992) to assess collinearity in the predictors.

Generalized linear models (GLMs; McCullagh & Nelder, 1989) were used to quantify the effect of fishing pressure and reef strata on adult *C. undulatus* and *B. muricatum* abundance. We chose GLMs with a negative binomial distribution and a log link because they are specifically designed for use with overdispersed count data containing a relatively high proportion of zeros (Dobson & Barnett, 2008). Subsequently, we identified the subset of predictor variables with the most support in the data from the full set of predictors (i.e., fishing pressure, reef strata, and mean‐centered latitude and longitude) using backward‐stepwise regression. The model with the smallest Akaike Information Criterion (AIC; Akaike, 1974) and root‐mean‐squared prediction error (RMSPE; Potts and Elith, 2006) based on the observations and the leave‐one‐out cross‐validation (LOOCV) was deemed the “best” model (Burnham & Anderson, 2004). The transect area varied across the 146 survey sites and so we also tested the need for transect area as an offset in the model (Table 1). An offset was included if the term's coefficient was significant at *α* = 0.05 or approximately equal to 1 in the full model. We assessed the goodness‐of‐fit for the final *C. undulatus* and *B. muricatum* models using half‐normal plots with simulated 95% confidence envelopes (Viera, Hinde, & Demetrio, 2000). We also calculated empirical semivariograms on the residuals from the two final models to check for spatial autocorrelation.

**Table 1:** Summary of *C. undulatus* and *B. muricatum* counts by reef strata and fishing pressure. Total survey area and number of surveys are also presented

Reef type	Fishing pressure	Survey area (m^2^)	Number of transects	Counts	
				*B. muricatum*	*C. undulatus*
Back reef	High	136,488	16	7	17
	Low	25,500	3	3	2
Fore reef	High	96,980	12	16	19
	Low	77,840	9	28	22
Fringing reef	High	79,072	14	3	6
	Low	89,878	14	10	9
Patch reef	High	40,614	6	1	11
	Low	270,250	35	40	43
Subtidal reef flat	High	6,660	1	0	0
	Low	341,060	36	211	59
Total			146	319	188

Spatial data were handled in R statistical software (R Core Team, 2016) using the raster (Hijmans, 2016), rgdal (Bivand, Keitt & Rowlingson, 2016) and sp (Pebesma and Bivand, 2005; Bivand, Pebesma & Gomez‐Rubio, 2013) packages and statistical analyses with the MASS (Venables & Ripley, 2002) and gstat (Pebesma, 2004) packages.

## 3 RESULTS

### 3.1 Graphical and numerical summaries

**Figure 2:**
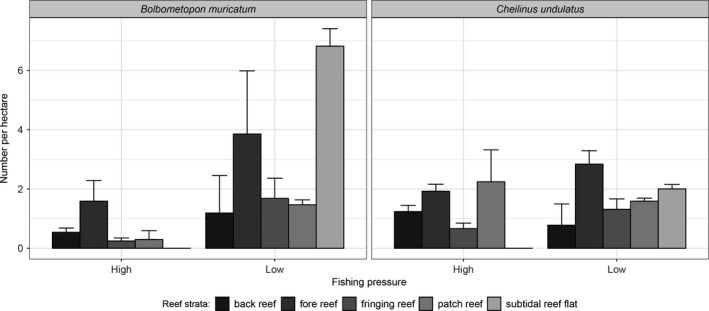
Average number of adult *B. muricatum* and *C. undulatus* per hectare by level of historical fishing pressure and reef strata. The error bars represent the upper bound of the 95% confidence intervals

Visual summaries suggested *B. muricatum* and *C. undulatus* responded differently to fishing pressure (Figure 2). For *B. muricatum*, mean densities across different reef strata were consistently lower in areas of high fishing pressure compared to areas with low fishing pressure. In contrast, the mean densities of *C. undulatus* did not vary significantly between areas of high and low fishing pressure. A numerical summary of the data (Table 1) showed the same trend between abundance, fishing pressure, and reef strata. Note that although the average densities of *C. undulatus* and *B. muricatum* are low (1.64 and 2.68 per hectare across all transects, respectively), this does not necessarily reflect densities within individual transects. The average densities are low because of the high proportion of transects with zero counts; 59 for *C. undulatus* and 92 for *B. muricatum*. Several individual transects had densities substantially higher than average. For *C. undulatus*, 58 transects had densities higher than the average and the highest density observed on one transect was 10 fish per hectare. For *B. muricatum*, a total of 30 transects had higher‐than‐average densities and the five highest densities were 40, 33.13, 32.86, 25.44, and 20.04 fish per hectare.

We assessed the potential predictor variables for collinearity and found all GVIF values were <1.9; except when longitude and latitude were included together, since these were inevitably correlated. Thus, collinearity in the predictors was not a significant issue in the models.

### 3.2 Final models

The final *C. undulatus* model included reef strata, latitude, longitude, and an interaction for latitude and longitude as predictor variables (Table 2, Model 2). The preferred reef strata for *C. undulatus* in decreasing order was fore reefs (i.e., reef slopes), back reefs, subtidal reef flats, and then patch reefs, with the lowest abundances found on fringing reefs (Table 3). In addition, there was a north–east to south–west gradient in adult *C. undulatus* abundance that was not explained by fishing pressure or reef strata. Fishing pressure was not included in the final *C. undulatus* model. When it was included, the effect of fishing pressure was small (e.g., areas of low fishing pressure only had 1.1 times more fish than areas of high pressure) and it was not statistically significant (*p*‐value = 0.7644; Table 2, Model 1).

**Table 2:** Final models for adult *C. undulatus* and *B. muricatum* abundance based on the Akaike Information Criterion (AIC) and the root mean‐squared‐prediction error (RMPSE) values generated by the observations and leave‐one‐out cross‐validation predictions. Models with the lowest AIC and RMSPE are shown in bold

Model ID	Model formula	*B. muricatum*		*C. undulatus*	
		AIC	RMSPE	AIC	RMSPE
1	Fishing pressure +	458.90	4.75	443.38	1.45
	Reef habitat type +				
	Latitude +				
	Longitude +				
	Latitude × Longitude				
2	Reef habitat type +	–	–	**441.48**	**1.44**
	Latitude +				
	Longitude +				
	Latitude × Longitude				
3	Fishing pressure +	458.66	4.66	–	–
	Reef habitat type +				
	Latitude +				
	Longitude				
4	Fishing pressure +	**456.67**	4.63	–	–
	Reef habitat type +				
	Latitude				
5	Fishing pressure +	457.11	**4.62**	–	–
	Reef habitat type +				
	Longitude				
6	None	478.93	5.04	459.71	1.53

**Table 3:** Parameter estimates and standard errors for the final *C. undulatus* and *B. muricatum* models. Effects significant at α = 0.05 are marked in bold. Estimates are only shown for predictors in the final model

Parameter	Estimate (S*E*)	
	*B. muricatum*	*C. undulatus*
Intercept (Fringing reef)	**−1.81 (0.60)**	−0.37 (0.31)
Low fishing pressure	**1.28 (0.50)**	–
Back reef	0.93 (0.75)	**1.01 (0.42)**
Fore reef	**1.95 (0.67)**	**1.68 (0.37)**
Patch reef	0.64 (0.61)	**0.76 (0.35)**
Subtidal reef	**2.39 (0.62)**	**1.16 (0.36)**
Latitude	−2.43 (1.36)	**−7.18 (1.88)**
Longitude	–	**−4.77 (1.55)**
Latitude × longitude	–	**20.39 (7.16)**

Two *B. muricatum* models were nearly identical in their AIC and RMSPE values, as well as their model coefficients. Both models contained reef strata and fishing pressure as predictors of abundance; one contained latitude and the other longitude (Table 2, Models 4 and 5). This result is not surprising given latitude and longitude were strongly correlated. Therefore, we selected Model 4 as the final *B. muricatum* model. The model results reflected the patterns we observed in the graphical summaries (Figure 2; Table 3). *B. muricatum* abundance was negatively affected by fishing pressure, with abundances in areas of low fishing pressure 3.6 times higher than in highly fished areas. *B. muricatum* abundance was also affected by reef strata; subtidal reef flats were the most preferred habitat, followed by fore reefs (i.e., reef slopes), while the lowest abundances were found in fringing, patch, and back reef strata (Table 3).

The GRTS survey design we used provided a spatially balanced sample stratified across the five reef strata. One advantage of GRTS is that it allocates more samples to reef strata with large areas, but also ensures that strata with small areas are sampled. As a result, only one transect was conducted on subtidal reef flats in areas of high fishing pressure. We were concerned that the disparity in transect numbers and abundances in subtidal reef flats in areas of low versus high fishing pressure (Table 1) might have influenced our results and so we refitted the models without data from subtidal reef flats. The results were virtually identical in their parameter estimates, standard errors, relative ordering of all other reef strata effects, and significance tests.

Half‐normal probability plots for the *B. muricatum* and *C. undulatus* showed each model fit the data reasonably well. There was no evidence of spatial autocorrelation in the model residuals. In addition, there was no evidence an offset for transect area was needed in any of the models.

## 4 DISCUSSION

Our findings highlight two points about the management of exploited fish populations in Central and Western Pacific reef systems. Firstly, even in closely related species, differences in habitat associations and behavior can heavily impact resilience to fishing pressure. Secondly, whenever possible, the pattern and scale of the sampling and data used to inform management decisions should match the scale at which they can be realistically applied. Local‐scale fisheries‐independent data can expose the responses of different species to the complex interactions of patterns of fish and human behavior; hence providing realistic and achievable goals relating to local cultural and economic conditions which may strongly affect fishing activities. In this Discussion, we first consider the biological features of *C. undulatus* and *B. muricatum* that determine their different responses to fishing pressure, and then consider the implications of our results for the management of fisheries and the appropriate data‐collection scale to inform those decisions.

### 4.1 Fishing pressure and vulnerability

Our results demonstrate that *B. muricatum* is more vulnerable to night spearfishing compared to *C. undulatus* in the Kia fishing grounds. *B. muricatum* abundances were 3.6 times lower in areas that had experienced high spearfishing pressure, while *C. undulatus* abundances were not influenced by spearfishing pressure. This was also true when we removed subtidal reef flats; *B. muricatum* abundances were 3.4 times lower in areas of high historical spearfishing pressure but had no effect on *C. undulatus*. Given that both *B. muricatum* and *C. undulatus* are very large fish that are relatively abundant in the Kia region, this observed difference warrants further discussion.

We believe the aggregation behavior of *B. muricatum* combined with the specific fishing strategies that were utilized in the Kia region are responsible for the differences we observed. Kia spearfishers use their extensive knowledge of their customary fishing grounds and *B. muricatum* nocturnal behavior to obtain high catches through a method known as spot checking (Hamilton et al., 2016). Fishers will travel to specific locations on the reef where B. muricatum schools are known to frequently form, then snorkel along the surface until they locate *B. muricatum* resting out in the open on the reef below them. If any are sighted, spearfishers free dive to spear the resting *B. muricatum*, then make a concerted effort to check the surrounding vicinity for other members of the school. If none are sighted, the fishers move on to another location where resting schools of *B. muricatum* are known to often reside. This spot checking method minimizes the amount free diving effort that is required; thus affording a degree of protection to more nocturnally cryptic species such as *C. undulatus* which do not aggregate at night and sleep within caves and crevices in the reef matrix; a nocturnal resting behavior that makes *C. undulatus* extremely difficult to sight from the surface.

Such spearfishing strategies are only effective in lightly fished areas where schools of *B. muricatum* can be reliably located. Indeed, in regions in Melanesia where *B. muricatum* populations have been depleted, night spearfishers adopt a different strategy, spending a considerable amount of their time continuously free diving and searching caves and crevices as they move along a reef (R.J. Hamilton, personal observation). As night spearfishers increasingly adopt an intensive free‐diving strategy, the likelihood of capturing more cryptic species such as *C. undulatus* increases. The use of SCUBA gear for night spearfishing will exacerbate this even further, as seen in Lindfield et al. (2014) where the catch composition of fisheries in Guam shifted dramatically toward *C. undulatus* when SCUBA was introduced.

Creel surveys of night spear fisheries that were conducted in lightly and moderately fished regions of the Western Solomon Islands in 2000–2001 provide some support for this explanation. Catch compositions from the lightly fished Tetepare Island consisted of 86% *B. muricatum* and 1.8% *C. undulatus*. This contrasts with the catch composition from the nearby, but more heavily fished Nusabanaga fishing grounds, where *B. muricatum* made up 56% of the catch and *C. undulatus* made up 5.6% of the catch (Hamilton, 2005).

Another possible explanation is that there is actually no difference in historical fishing pressure between zones in the Kia fishing grounds and the difference in abundance exists for other reasons. However, this is unlikely since the fishermen interviewed had vast knowledge of fishing practices around Kia (Hamilton et al., 2016). They unanimously identified Zone 1 as the zone of high historical fishing pressure, citing significant and tangible factors like proximity to the BPFC, fuel costs, and issues around customary fishing rights.

### 4.2 Habitat preferences

We included reef strata in the models to account for natural variation in *B. muricatum* and *C. undulatus* abundance. Our results agree with qualitative reports from previous studies, and provide novel information that can be linked to the ecology of these fishes. Adult *B. muricatum* preferred subtidal reefs and fore reefs as habitats, with higher abundances in these areas relative to back and patch reefs. The preference of *B. muricatum* for subtidal reef flats is likely a function of their feeding behavior, as this species feeds in exposed areas where protein‐rich endolithic organisms (e.g., cyanobacteria and filamentous green algae) are abundant and benthic organisms like corals and macroscopic algae are comparatively less so (Clements, German, Piche, Tribollet, & Choat, 2017; Donaldson & Dulvy, 2004; Hoey & Bellwood, 2008). In contrast, adult *C. undulatus* preferred fore reefs, where the abundance was 5.4 times higher compared to fringing reefs (the least preferred) and 1.7 times higher compared to subtidal reefs. This is likely related to its reliance on complex reef habitats for feeding and sheltering at night (Donaldson & Sadovy, 2001; Sadovy et al., 2003).

### 4.3 Management implications

The difference we found in the vulnerabilities of *B. muricatum* and *C. undulatus* to night spearfishing pressure in the lightly exploited Kia region may be surprising to some, given *C. undulatus* is listed as endangered on the IUCN Red List of Threatened Species and there are protections against international trade under CITES (Vincent et al., 2014). In addition, previous analyses of UVC survey data from New Caledonia and French Polynesia, as well as a dataset collected across the geographic range of *C. undulatus*, found fishing pressure correlates to serious declines in *C. undulatus* abundance*,* with 10‐fold decreases in densities in fished areas compared to unfished areas (Lavides et al., 2016; Sadovy et al., 2003). We believe that the results we found differ to those of previous studies because of differences in scale. Declines in both *B. muricatum* and *C. undulatus* are regularly attributed to excess fishing pressure (Fenner, 2014) and this is likely true at a global scale, across all types of fishing (e.g., spearfishing, hook‐and‐line, nets). Information about drivers of species decline at this scale are also based on data aggregated across areas with a range of human population density, natural habitats, and reef types. However, our results demonstrate that coarse‐scale information about species vulnerability and its potential causes does not necessarily capture drivers of vulnerability at the local (i.e., meters to thousands of hectares; Poiani, Richter, Anderson, & Richter, 2000) or subpopulation scale. This is not surprising given the variation in fishing methods and relative level of pressure at a global or even national scale; especially for heavily populated areas.

There is evidence for scale‐dependence in the effects of anthropogenic fishing pressure. In a study of parrotfish assemblages including *B. muricatum* across eight Micronesian islands, Taylor, Lindfield, and Choat (2014) found that island geomorphology and species distribution patterns accounted for the greatest variation in assemblage structure, species richness and diversity at biogeographic scales. In contrast, the effects of fishing pressure were only strongly expressed at the within‐island scale.

There are also numerous examples where a combination of local harvesting practices and the behavior of natural populations has led to extinctions and/or shifts in the spatial distribution of a species. One famous example is the northwest cod (*Gadus morhua*) fishery, which was rapidly depleted when trawling was introduced because schools of spawning fish could be caught more efficiently than with hook‐and‐line methods (Ames, 2004). Another example is found in the Bay of Martaban, Myanmar, where nearly half of all spoon‐billed sandpipers (*Calidris pygmaea*) winter. The increased use of mist nets by bird catchers has significantly increased sandpiper by‐catch and hunters in this poor community are likely to eat or sell the birds, rather than release them (Zockler et al., 2010). In this case, the combination of bird behavior, the decreasing cost of mist nets, and the low economic status of the local community has led the species to the brink of extinction. Cronin et al. (2016) also found evidence that two of seven monkey species, *Cercopithecus erythrotis* and *C. nictitans,* were resilient to relatively high gun‐hunting pressure in Bioko Island, Equatorial Guinea; a finding which was attributed to elevated anti‐predator behavior, smaller group sizes, ecological flexibility, and life‐history traits. In each case, the effective management of the species in question depended on local‐scale information about the interactions between humans, their hunting methods, and the species’ natural behaviors.

The clear management implication of this study is that local‐scale assessments within specific fisheries (or other) contexts can help to identify instances where the natural behavior of a species makes them vulnerable before an unexpected population crash occurs. Therefore, the fundamental question becomes: which species requires the most urgent attention, given the level of harm they are biologically predisposed to as a result of the predominant fishing or hunting practices? If the answer is based solely on global vulnerability studies or catch‐dependent data, the resulting conservation outcomes may be unexpected and undesirable. By the same token, the relative local‐scale vulnerabilities of species in one region (e.g., *B. muricatum* and *C. undulatus* in Kia) are not necessarily transferable to other areas because differences in human population densities, harvesting practices and natural habitat compositions, as well as community compositions can lead to different results (e.g., Lindfield et al., 2014).

For the Kia fishery, the subpopulation structure of adult fishes and their responses to fisheries are differentiated at much finer spatial scales than have been previously considered for management efforts. Thus, our results suggests *B. muricatum* is in need of more attention in Kia; even though *C. undulatus* is allocated a higher conservation priority at the global scale. While this observation itself is novel, we also demonstrate that a mismatch in scale occurs between the information and its application in decision‐making when global‐scale assessments of vulnerability are exclusively used to inform local‐scale management decisions; as a result, important phenomena such as the extreme vulnerability of *B. muricatum* to night spearfishing may be missed. However, our findings do not imply that *C. undulatus* are not susceptible to fishing or their current global conservation status is unwarranted. For example, there is still evidence of cyanide fishing for this species within the Coral Triangle, although this is now illegal (Gillett, 2010; Sadovy et al., 2003). Spearfishing on SCUBA may also pose a serious threat to *C. undulatus* as this technology becomes more widespread in the Indo‐Pacific, although this method is also illegal in many areas (Lindfield et al., 2014). Furthermore, both species have been rapidly depleted in areas of the Indo‐Pacific with much higher human densities than the Kia fishing grounds (Lavides et al., 2016). Additionally, in terms of global‐scale conservation actions, the regulations imposed by CITES on the international trade of *C. undulatus* (Vincent et al., 2014) are instrumental, both symbolically and materially, in complementing local and regional strategies to prevent the extinction of the species. We have no intention of undoing this work. Nevertheless, our results clearly show that local‐scale variability exists in the relative level of vulnerability of *B. muricatum* and *C. undulatus* to night spearfishing without SCUBA in Kia. These local‐scale differences in species vulnerability may also play an important role in other regions and should be assessed using fisheries‐independent data, where resources permit.

## 5 CONCLUSIONS

We hope our findings will help to build a clearer picture of the extreme vulnerability of *B. muricatum* to the common practice of night spearfishing and, in so doing, help to drive additional national and regional protection and global awareness for this species. We must reiterate, however, that our findings at Kia, with its specific habitats, local population and fishing methods, should not detract from the attention given to *C. undulatus* globally and in other parts of the Indo‐Pacific. To draw conservation resources away from *C. undulatus* in other fisheries based on our results from Kia would be contrary to the main point of this paper, where we have demonstrated the need for local‐scale, fisheries‐independent data to inform management decisions at relevant scales. The historical variations in the intensity of fishing across space and time, the method of fishing employed, the natural behavioral vulnerabilities of certain species that such methods exploit, and the local distribution of fishes throughout different habitats, are factors that will vary intensely at fine scales. Hence, our results regarding these specific fishes may not be reliably generalized for other fisheries. Instead, we call on regional managers to carefully assess the state of their fisheries and the dynamics affecting the vulnerability of the fishes therein to fishing pressure based on local‐scale, fisheries‐independent data to better allocate conservation resources.

## AUTHOR CONTRIBUTION

R.J.H., J.P., G.R.A., and N.A.P. collected the data. A.R.P., E.E.P., and G.S.H. analyzed the data. A.R.P., R.J.H, J.H.C., and E.E.P. interpreted the results. A.R.P. wrote the manuscript with contributions from the other authors; most significantly from R.J.H., J.H.C., and E.E.P.

## DATA ACCESSIBILITY

Our transect data for *C. undulatus* and *B. muricatum* abundance, as well as shapefiles of the fishing zones and reef strata around Kia, are available on Dryad (https://doi.org/10.5061/dryad.pg440kd).

## References

[ece34501-bib-0001] Akaike, H. (1974). A new look at the statistical model identification. IEEE Transactions on Automatic Control, 19(6), 716–723. 10.1109/TAC.1974.1100705

[ece34501-bib-0002] Ames, E. P. (2004). Atlantic cod stock structure in the Gulf of Maine. Fisheries, 29(1), 10–28. 10.1577/1548-8446(2004)29[10:ACSSIT]2.0.CO;2

[ece34501-bib-0003] Andrefouet, S. , Muller‐Karger, F. E. , Robinson, J. A. , Kranenburg, C. J. , Torres‐Pulliza, D. , Spraggins, S. A. , & Murch, B. (2006). Global assessment of modern coral reef extent and diversity for regional science and management applications: a view from space. *Proceedings of the 10th International Coral Reef Symposium*, 2, 1732–1745.

[ece34501-bib-0004] Andrews, A. H. , Choat, J. H. , Hamilton, R. J. , & DeMartini, E. E. (2015). Refined bomb radiocarbon dating of two iconic fishes of the Great Barrier Reef. Marine and Freshwater Research, 66(4), 305–316. 10.1071/MF14086

[ece34501-bib-0005] Bellwood, D. R. , Hoey, A. S. , & Choat, J. H. (2003). Limited functional redundancy in high diversity systems: Resilience and ecosystem function on coral reefs. Ecology Letters, 6, 281–285. 10.1046/j.1461-0248.2003.00432.x

[ece34501-bib-0061] Bivand, R. , Keitt, T. , & Rowlingson, B. (2016). rgdal: Bindings for the Geospatial Data Abstraction Library. R package version 1.1‐10. https://CRAN.R-project.org/package=rgdal

[ece34501-bib-0062] Bivand, R. , Pebesma, E. , & Gomez‐Rubio, V. (2013). Applied spatial data analysis with R, 2nd ed. New York, NY: Springer.

[ece34501-bib-0006] Burnham, K. P. , & Anderson, D. R. (2004). Multimodel inference: Understanding AIC and BIC in model selection. Sociological Methods and Research, 33, 261–304. 10.1177/0049124104268644

[ece34501-bib-0007] Choat, J. H. , Davies, C. R. , Ackerman, J. L. , & Mapstone, B. D. (2006). Age structure and growth in a large teleost, Cheilinus undulatus, with a review of size distribution in labrid fishes. Marine Ecology Progress Series, 318, 237–246. 10.3354/MEPS318237

[ece34501-bib-0008] Choat, J. H. , & Pears, R. (2003). A rapid, quantitative survey method for large, vulnerable reef fishes In Wilkinson C., Green A., Almany J. & Dionne S. (Eds.), Monitoring coral reef marine protected areas. A practical guide on how monitoring can support effective management MPAs (pp. 54–55). Townsville: Australian Institute of Marine Science and the IUCN Marine Program Publication.

[ece34501-bib-0009] Clements, K. D. , German, D. P. , Piche, J. , Tribollet, A. , & Choat, J. H. (2017). Integrating ecological roles and trophic diversification on coral reefs: Multiple lines of evidence identify parrotfishes as microphages. Biological Journal of the Linnaean Society, 120(4), 729–751. 10.1111/bij.12914

[ece34501-bib-0010] Colin, P. L. , & Sadovy de Mitcheson, Y. (2012). Humphead wrasse – *Cheilinus undulatus* In de Mitcheson Y. Sadovy, & Colin P. L. (Eds.), Reef fish spawning aggregations: Biology, research and management (Vol. 35, pp. 478–487). Dordrecht: Springer Fish and Fisheries Series, Springer Science + Business Media 10.1007/978-94-007-1980-4

[ece34501-bib-0011] Cronin, D. T. , Riaco, C. , Linder, J. M. , Bergl, R. A. , Gonder, M. K. , O'Connor, M. P. , & Hearn, G. W. (2016). Impact of gun‐hunting on monkey species and implications for primate conservation on Bioko Island, Equatorial Guinea. Biological Conservation, 197, 180–189. 10.1016/j.biocon.2016.03.001

[ece34501-bib-0012] Dobson, A. J. , & Barnett, A. G. (2008). An introduction to generalised linear models (3rd ed.). London, UK: Chapman and Hall.

[ece34501-bib-0013] Donaldson, T. J. , & Dulvy, N. K. (2004). Threatened fishes of the world: Bolbometopon muricatum (valenciennes 1840) (scaridae). Environmental Biology of Fishes, 70(4), 373 10.1023/B:EBFI.0000035509.89614.1e

[ece34501-bib-0014] Donaldson, T. J. , & Sadovy, Y. (2001). Threatened fishes of the world: *Cheilinus undulatus* Ruppell, 1835 (Labridae). Environmental Biology of Fishes, 62, 428 10.1023/A:1012221911224

[ece34501-bib-0015] Dulvy, N. K. , & Polunin, N. V. C. (2004). Using informal knowledge to infer human‐induced rarity of a conspicuous reef fish. Animal Conservation, 7, 365–374. 10.1017/S1367943004001519

[ece34501-bib-0016] Fenner, D. (2014). Fishing down the largest coral reef fish species. Marine Pollution Bulletin, 84, 9–16. 10.1016/j.marpolbul.2014.04.049 24889317

[ece34501-bib-0017] Fox, J. , & Monette, G. (1992). Generalized collinearity diagnostics. Journal of the American Statistical Association, 87, 173–183.

[ece34501-bib-0018] Gillett, R. (2010). Monitoring and management of the humphead wrasse, Cheilinus undulatus. *FAO Fisheries and Aquaculture Circular* No. 1048. Rome, FAO. 2010. 62 p.

[ece34501-bib-0019] Gillett, R. , & Moy, W. (2006). Spearfishing in the Pacific Islands. Current Status and Management Issues. FAO/FishCode Review No. 19. FAO, Rome.

[ece34501-bib-0020] Green, A. L. , Maypa, A. P. , Almany, G. R. , Rhodes, K. L. , Weeks, R. , Abesamis, R. A. , … White, A. T. (2015). Larval dispersal and movement patterns of coral reef fishes, and implications for marine reserve network design. Biological Reviews, 90, 1215–1247. 10.1111/brv.12155 25423947

[ece34501-bib-0021] Hamilton, R. J. (2005). The demographics of Bumphead Parrotfish (Bolbometopon muricatum) in lightly and heavily fished regions of the Western Solomon Islands. Ph.D. Thesis, University of Otago, Dunedin, New Zealand, p. 273.

[ece34501-bib-0022] Hamilton, R. J. , Almany, G. R. , Brown, C. J. , Pita, J. , Peterson, N. A. , & Choat, J. H. (2017). Logging degrades nursery habitat for an iconic coral reef fish. Biological Conservation, 210(Part A), 273–280. 10.1016/j.biocon.2017.04.024

[ece34501-bib-0023] Hamilton, R. J. , Almany, G. R. , Stevens, D. , Bode, M. , Pita, J. , Peterson, N. A. , & Choat, J. H. (2016). Hyperstability masks declines in bumphead parrotfish (*Bolbometopon murricatum*) populations. Coral Reefs, 35(3), 751–763. 10.1007/s00338-016-1441-0

[ece34501-bib-0024] Hamilton, R. J. , & Choat, J. H. (2012). Bumphead parrotfish: *Bolbometopon muricatum* In de Mitcheson Y. Sadovy, & Colin P. L. (Eds.), Reef fish spawning aggregations: Biology, research and management, Vol. 35 (pp. 490–496). Dordrecht: Springer Fish and Fisheries Series, Springer Science + Business Media.

[ece34501-bib-0025] Hamilton, R. J. , Giningele, M. , Aswani, S. , & Ecochard, J. L. (2012). Fishing in the dark—local knowledge, night spearfishing and spawning aggregations in the Western Solomon Islands. Biological Conservation, 145(1), 246–257. 10.1016/j.biocon.2011.11.020

[ece34501-bib-0063] Hijmans, R.J. (2016). raster: Geographic data analysis and modeling. R package version 2.5‐8. https://CRAN.R-project.org/package=raster

[ece34501-bib-0026] Hoey, D. S. , & Bellwood, D. R. (2008). Cross‐shelf variation in the role of parrotfishes on the Great Barrier Reef. Coral Reefs, 27(1), 37–47. 10.1007/s00338-007-0287-x

[ece34501-bib-0027] Hutchings, J. A. (1996). Spatial and temporal variation in the density of northern cod and a review of hypotheses for the stock's collapse. Canadian Journal of Fisheries and Aquatic Sciences, 53(5), 943–962. 10.1139/f96-097

[ece34501-bib-0028] Kindsvater, H. K. , Reynolds, J. D. , Savody de Mitcheson, Y. , & Mangel, M. (2017). Selectivity matters: Rules of thumb for management of plate‐sized, sex‐changing fish in the live reef food fish trade. Fish and Fisheries, 18(5), 821–836. 10.1111/faf.12208

[ece34501-bib-0065] Kobayashi, D. , Friedlander, A. , Grimes, C. , Nichols, R. , & Zgliczynski, B. (2011). Bumphead parrotfish (Bolbometopon muricatum) status review. NOAA Technical Memorandum NMFS‐PIFSC‐26, Pacific Islands Fisheries Science Center, Hawaii, p. 102

[ece34501-bib-0029] Lavides, M. N. , Molina, E. P. V. , de la Rosa, G. E. , Mill, A. C. , Rushton, S. P. , Stead, S. M. , & Polunin, N. V. C. (2016). Patterns of Coral‐Reef Finfish Species Disappearances Inferred from Fishers’ Knowledge in Global Epicentre of Marine Shorefish Diversity. PLoS ONE, 11(5), e0155752 10.1371/journal.pone.0155752 27191602 PMC4871521

[ece34501-bib-0030] Lindfield, S. J. , McIlwain, J. L. , & Harvey, E. S. (2014). Depth Refuge and the Impacts of SCUBA Spearfishing on Coral Reef Fishes. PLoS ONE, 9(3), e92628 10.1371/journal.pone.0092628 24663400 PMC3963921

[ece34501-bib-0031] McCullagh, P. , & Nelder, J. A. (1989). Generalised linear models. Monographs on statistics and applied probability. London, UK: Chapman and Hall.

[ece34501-bib-0032] Pebesma, E. J. (2004). Multivariable geostatistics in S: The gstat package. Computers & Geosciences, 30, 683–691. 10.1016/j.cageo.2004.03.012

[ece34501-bib-0067] Pebesma, E. J. , & Bivand, R. (2005). Classes and methods for spatial data in R. R News, 5(2), 20–41.

[ece34501-bib-0033] Peterson, N. , Hamilton, R. , Pita, J. , Atu, W. , & James, R. (2012). Ridges to reefs conservation plan for Isabel Province, Solomon Islands. Brisbane, QLD: The Nature Conservancy, Indo‐Pacific Division, Solomon Islands.

[ece34501-bib-0034] Poiani, K. A. , Richter, B. D. , Anderson, M. G. , & Richter, H. E. (2000). Biodiversity conservation at multiple scales: Functional sites, landscapes and networks. BioScience, 50(2), 133–146. 10.1641/0006-3568(2000)050[0133:BCAMSF]2.3.CO;2

[ece34501-bib-0035] Potts, J. M. , & Elith, J. (2006). Comparing species abundance models. Ecological modelling, 199, 153–163. 10.1016/j.ecolmodel.2006.05.025

[ece34501-bib-0036] R Core Team (2016). R: A language and environment for statistical computing. R Foundation for Statistical Computing, Vienna, Austria. Retrieved from https://www.R-project.org/

[ece34501-bib-0037] Sadovy de Mitcheson, Y. , & Erisman, B. E. (2012). The social and economic importance of aggregating species and the biological implications of fishing on spawning aggregations In Sadovy de Mitcheson Y. & Colin P. (Eds.), Reef fish spawning aggregations: Biology, research and management. Dordrecht: Springer 10.1007/978-94-007-1980-4

[ece34501-bib-0038] Sadovy, Y. , & Domeier, M. (2005). Are aggregation‐fisheries sustainable? Reef fish fisheries as a case study. Coral Reefs, 24, 254–262. 10.1007/s00338-005-0474-6

[ece34501-bib-0039] Sadovy, Y. , Kulbicki, M. , Labrosse, P. , Letourneur, Y. , Lokani, P. , & Donaldson, T. J. (2003). The humphead wrasse, cheilinus undulatus: Synopsis of a threatened and poorly known giant coral reef fish. Reviews in Fish Biology and Fisheries, 13(3), 327–364. 10.1023/B:RFBF.0000033122.90679.97

[ece34501-bib-0040] Stevens, D. L. , & Olsen, A. R. (2004). Spatially balanced sampling of natural resources. Journal of the American Statistical Association, 99(465), 262–278. https://doi.org/10/1198/01621450400000250

[ece34501-bib-0041] Taylor, B. M. , Lindfield, S. J. , & Choat, J. H. (2014). Hierarchical and scale‐dependent effects of fishing pressure and environment on the structure and size distribution of parrotfish communities. Ecography, 37, 1–11. 10.1111/ecog.01093

[ece34501-bib-0042] Venables, W. N. , & Ripley, B. D. (2002). Modern applied statistics with S (4th ed.). New York, NY: Springer 10.1007/978-0-387-21706-2

[ece34501-bib-0043] Viera, A. M. C. , Hinde, J. P. , & Demetrio, C. G. B. (2000). Zero‐inflated proportion data models applied to a biological control assay. Journal of Applied Statistics, 27(3), 373–389. 10.1080/02664760021673

[ece34501-bib-0044] Vincent, A. C. J. , Sadovy de Mitcheson, Y. J. , Fowler, S. L. , & Lieberman, S. (2014). The role of CITES in the conservation of marine fishes subject to international trade. Fish and Fisheries, 15, 563–592. 10.1111/faf.12035

[ece34501-bib-0045] Westneat, M. W. , & Alfaro, M. E. (2005). Phylogenetic relationships and evolutionary history of the reef fish family Labridae. Molecular Phylogenetics and Evolution, 36, 370–390. 10.1016/j.ympev.2005.02.001 15955516

[ece34501-bib-0046] Zgliczynski, B. J. , Williams, I. D. , Schroeder, R. E. , Nadon, M. O. , Richards, B. L. , & Sandin, S. A. (2013). The IUCN Red List of Threatened Species: An assessment of coral reef fishes in the US Pacific Islands. Coral Reefs, 32, 637–650. 10.1007/s00338-013-1018-0

[ece34501-bib-0047] Zockler, C. , Hla, T. H. , Clark, N. , Syroechkovskiy, E. , Yakushev, N. , Daengphayon, S. , & Robinson, R. (2010). Hunting in Myanmar is probably the main cause of decline in the Spoon‐billed Sandpiper Calidris pygmeus. Wader Study Group Bulletin, 117(1), 1–8.

