# Supplementary material for: Giant coral reef fishes display markedly different susceptibility to night spearfishing
Source: Ecol Evol. 2018 Sep 12;8(20):10247–56. doi: 10.1002/ece3.4501 (PMC6206199; doi:10.1002/ece3.4501)
Supplement: Supplementary file 1 [file ECE3-8-10247-s001.docx]

**Supplementary information A: classification of reef strata based on Millennium Coral Reef Mapping attributes**

The reef strata used in this study were derived from the Millennium Coral Reef Mapping (MCRM) data (Andrefouet et al., 2006). This dataset contains hierarchical classifications of reef geomorphologies using Landsat 8 images at four levels: L1 (coarsest) to L4 (finest). We began by aggregating the L3 and L4 attributes (Table A1). We then compared the new combined classes with satellite images and our own local knowledge of the area before reclassing them into the 5 reef strata classes.

Table A1. Final classifications of reef strata based on aggregated Level 3 and Level 4 attributes from the Millennium Coral Reef Mapping project.

| **L3 code** | **L3 attribute** | **L4 code** | **L4 attribute** | **Final classification** |
| --- | --- | --- | --- | --- |
| 16 | Outer Barrier Reef Complex | 48 | reef flat | back reef |
| 16 | Outer Barrier Reef Complex | 30 | forereef | fore reef |
| 27 | Ocean exposed fringing | 30 | forereef | fringing reef |
| 27 | Ocean exposed fringing | 48 | reef flat | fringing reef |
| 28 | Intra-seas exposed fringing | 30 | forereef | fringing reef |
| 28 | Intra-seas exposed fringing | 48 | reef flat | fringing reef |
| 29 | Lagoon exposed fringing | 30 | forereef | fringing reef |
| 29 | Lagoon exposed fringing | 48 | reef flat | fringing reef |
| 22 | Coastal/fringing patch | 48 | reef flat | patch reef |
| 24 | Intra-lagoon patch-reef complex | 48 | reef flat | patch reef |
| 24 | Intra-lagoon patch-reef complex | 63 | subtidal reef flat | patch reef |
| 25 | Intra-seas patch-reef complex | 30 | forereef | patch reef |
| 25 | Intra-seas patch-reef complex | 48 | reef flat | patch reef |
| 25 | Intra-seas patch-reef complex | 63 | subtidal reef flat | patch reef |
| 16 | Outer Barrier Reef Complex | 63 | subtidal reef flat | subtidal reef flat |

**References**

Andrefouet, S., Muller-Karger, F. E., Robinson, J. A., Kranenburg, C. J., Torres-Pulliza, D., Spraggins, S. A., and Murch, B. (2006). Global assessment of modern coral reef extent and diversity for regional science and management applications: a view from space. *Proceedings of the 10^th^ International Coral Reef Symposium,­­­ 2,* 1732 – 1745.
